# Robotic-assisted thoracoscopic surgery for esophageal duplication cyst in a child: a rare case report and literature insight

**DOI:** 10.3389/fped.2025.1679015

**Published:** 2025-10-03

**Authors:** Boshen Shu, Shufeng Zhang, Jian Gao, Lin Wang, Xiaohui Wang

**Affiliations:** Department of Pediatric Surgery, Henan Provincial People’s Hospital, Zhengzhou, Henan, China

**Keywords:** esophageal duplication cyst, child, congenital, robotic surgery, thoracoscopy, case report

## Abstract

**Introduction:**

Esophageal duplication cyst (EDC) is a rare congenital anomaly originating from the embryonic foregut. While often asymptomatic, it can present with respiratory symptoms due to compression. We report a rare pediatric case successfully managed using robotic-assisted thoracoscopic surgery (RATS), highlighting the technique's utility in addressing this uncommon foregut malformation.

**Presentation of case:**

An 8-year-old male child, born at term, presented with one week of intermittent left-sided chest pain, bloating, and nausea. Chest radiography demonstrated a high-density mediastinal shadow. Subsequent chest positron emission tomography/computed tomography (PET/CT) and ultrasound (US) revealed a left posterior mediastinal paraspinal cystic mass, necessitating surgical resection. The lesion was diagnosed as an esophageal duplication cyst based on the post-operative pathological report.

**Discussion:**

EDC is a rare congenital condition that can become life-threatening due to compression of vital mediastinal structures, particularly the large airways. Non-invasive imaging modalities, including chest radiography, CT, and US, are essential for diagnosis. Surgical intervention is universally indicated to preempt serious complications. RATS has recently emerged as a promising approach, leveraging its technical advantages for this complex procedure.

**Conclusion:**

To the best of our knowledge, the present case represents a unique and extremely rare report of EDC management via the Da Vinci robotic surgical system for a pediatric patient in China. The successful application demonstrates the safety and feasibility of RATS for pediatric EDC, expanding the evidence base for this minimally invasive approach.

## 1 Introduction

EDC, a rare congenital anomaly originating from the embryonic foregut, encompassing both bronchopulmonary and alimentary tract lineages. Clinical manifestations typically arise from mass effect secondary to cystic enlargement. In children, common manifestations include stridor, dyspnea, wheezing, and dysphagia, whereas neonates more frequently exhibit overt respiratory distress (1). As rare congenital malformations, EDC constitutes 0.5% to 2.5% of all esophageal masses (2). Epidemiological data estimate an incidence of 1 in 8,200 live births, with a reported male-to-female prevalence ratio of 2:1 (3). While CT and US are valuable imaging tools for evaluation, definitive diagnosis of EDC requires pathological confirmation and surgical intervention is advisable for both symptomatic and asymptomatic patients, considering the potential risks of subsequent complications and malignant transformation (4, 5).

In this report, we present the successful application of RATS for EDC resection in an 8-year-old boy. Compared to traditional open thoracotomy, RATS offers significant advantages, including smaller incisions, free rotation of the robotic arm, accelerated recovery, and lower complication rates (6, 7). To the best of our knowledge, this case represents an exceptionally rare report of EDC management using the Da Vinci robotic surgical system in a Chinese pediatric patient. It thereby may address a research gap regarding the application of robotic-assisted surgery for treating pediatric EDC. This case report adheres to the SCARE guidelines (8).

## 2 Case presentation

### 2.1 Patient information

**Figure 1:**
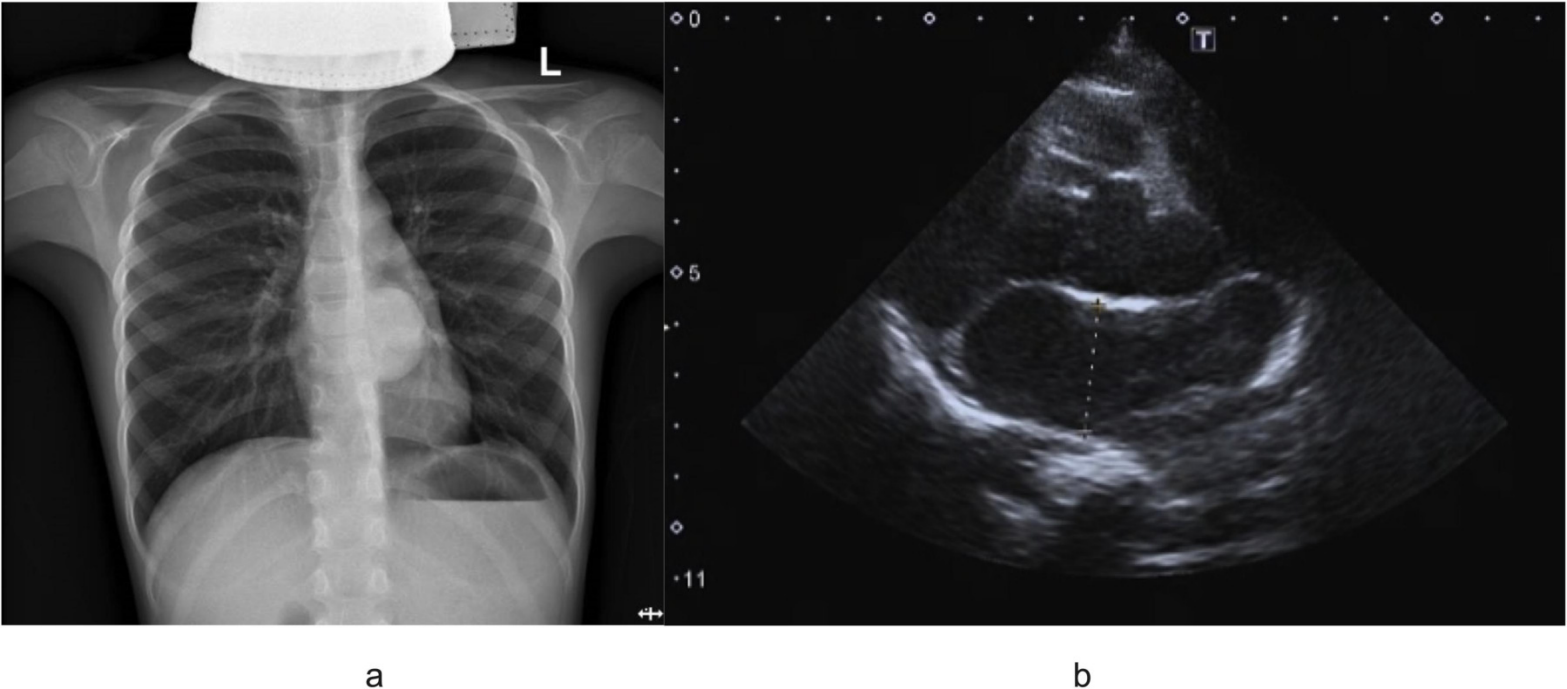
**(a)** Outcomes of preoperative chest radiograph demonstrated a high-density mediastinal shadow; **(b)** preoperative ultrasound examination indicated left posterior mediastinal mass lesion.

**Figure 2:**
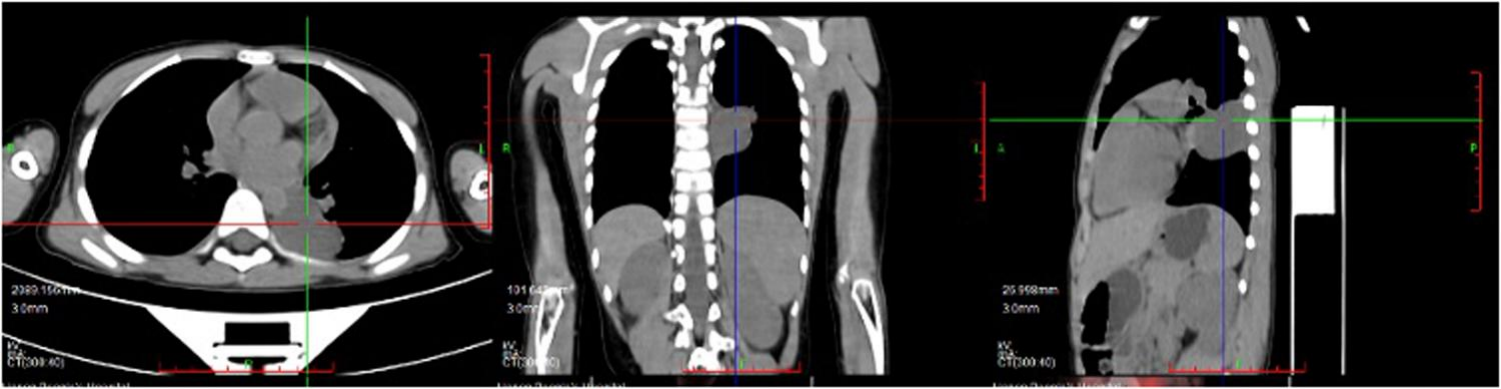
Preoperative PET/CT indicated paraspinal cystic lesion in the left lower lobe with loss of metabolism.

An 8-year-old boy presented with a one-week history of intermittent left-sided chest pain, bloating, and nausea. On physical examination, all findings were within normal ranges except for left chest tenderness and slightly restricted respiratory movements. Preoperative imaging included chest radiography, US, and PET/CT. The chest radiograph demonstrated a high-density mediastinal shadow, while US identified a 55*48*37 mm posterior mediastinal mass in the suprasternal region (Figure 1). Subsequent PET/CT further characterized a 37*31*37 mm paraspinal cystic lesion in the left lower lobe with loss of metabolism, confirming the need for surgical resection (Figure 2). Laboratory studies indicated no contraindications to surgery.

### 2.2 Robot-assisted surgery

**Figure 3:**
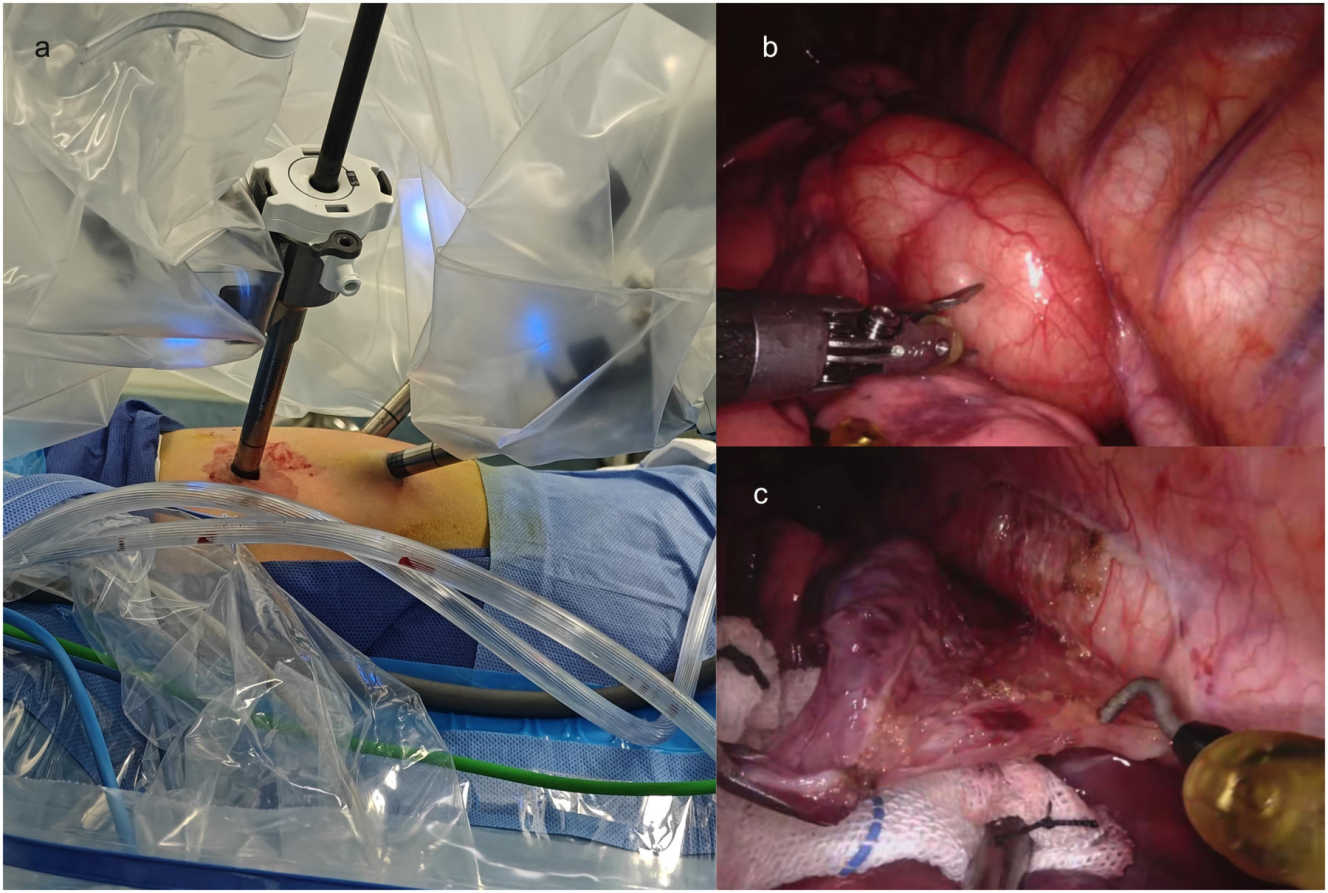
Intraoperative findings and dissection of a mediastinal chylous cyst. **(a)** The placement of Trocars; **(b)** Thoracic cavity exploration revealing a 60 × 50 × 40 mm mediastinal cyst filled with chylous fluid, densely adherent to the esophagus, thoracic aorta, and left lower lobe; **(c)** Meticulous dissection of the cyst using an electrosurgical hook and microbipolar forceps to release adhesions.

Given concerns regarding potential inflammatory bronchospasm and mass compression effects, preoperative medications were initiated with cefazolin (antibiotic prophylaxis), budesonide (inhaled corticosteroid), terbutaline (*β*₂-agonist), and ambroxol (mucolytic). The patient underwent RATS for cyst resection, with the technical approach detailed below. Following successful induction of general anesthesia, the child was positioned in the right lateral decubitus posture. Routine skin preparation and draping were performed. A 2-cm incision was made at the 8th intercostal space along the anterior axillary line, through which a single-port laparoscopic device was inserted, and the thoracoscope was connected. Two additional 8-mm robotic trocars were inserted under direct thoracoscopic vision: one in the 6th intercostal space and another in the 10th intercostal space along the anterior axillary line (Figure 3a).

Thoracic cavity exploration revealed a 60*50*40 mm mediastinal cyst containing chylous fluid, which was closely adherent to the esophagus, thoracic aorta, and left lower lobe (Figure 3b). Specifically, the cyst was intimately associated with the esophageal wall: its outer capsule shared a thin, fibrous plane with the esophageal muscularis propria, with no invasion into the submucosa or esophageal lumen. Notably, the adherence was most dense at the cyst's posterior aspect, where it was embedded within the outer third of the esophageal muscle fibers, consistent with the congenital nature of EDC, where abnormal foregut budding leads to intimate but non-invasive attachment to the native esophageal wall. This anatomical relationship required meticulous dissection using an electrosurgical hook and microbipolar forceps to preserve the integrity of the underlying esophageal mucosa, which remained intact throughout the procedure (as confirmed by intraoperative inspection) (Figure 3c).

The cyst was completely resected at its base, and following excision, a small defect (approximately 4 mm in diameter) was identified in the esophageal muscularis propria, corresponding to the area where the cyst had been most tightly adherent. Given the pediatric patient's need for unobstructed esophageal growth and function, repair was performed using 5-0 absorbable polyglactin sutures (Vicryl) in an interrupted, tension-free manner. The sutures were placed to approximate the muscular edges along the longitudinal axis of the esophagus, avoiding circumferential closure to prevent luminal narrowing, a key consideration in pediatric patients with growing digestive tracts. The repair was reinforced with a small patch of adjacent mediastinal fat (harvested from the surgical field) to buttress the muscular defect, secured with 6-0 absorbable sutures. Intraoperative inspection confirmed no mucosal injury, and the esophageal lumen remained patent without evidence of narrowing.

The resected cyst was submitted for pathological examination. The operative field was irrigated with warm saline, with no active bleeding or air leak observed. After verifying correct counts of instruments and supplies, the incisions were sutured layer by layer. A drainage tube was placed through the 10th intercostal space at the posterior axillary line, and the incision was closed with intermittent sutures. The procedure was completed successfully.

## 3 Results

**Figure 4:**
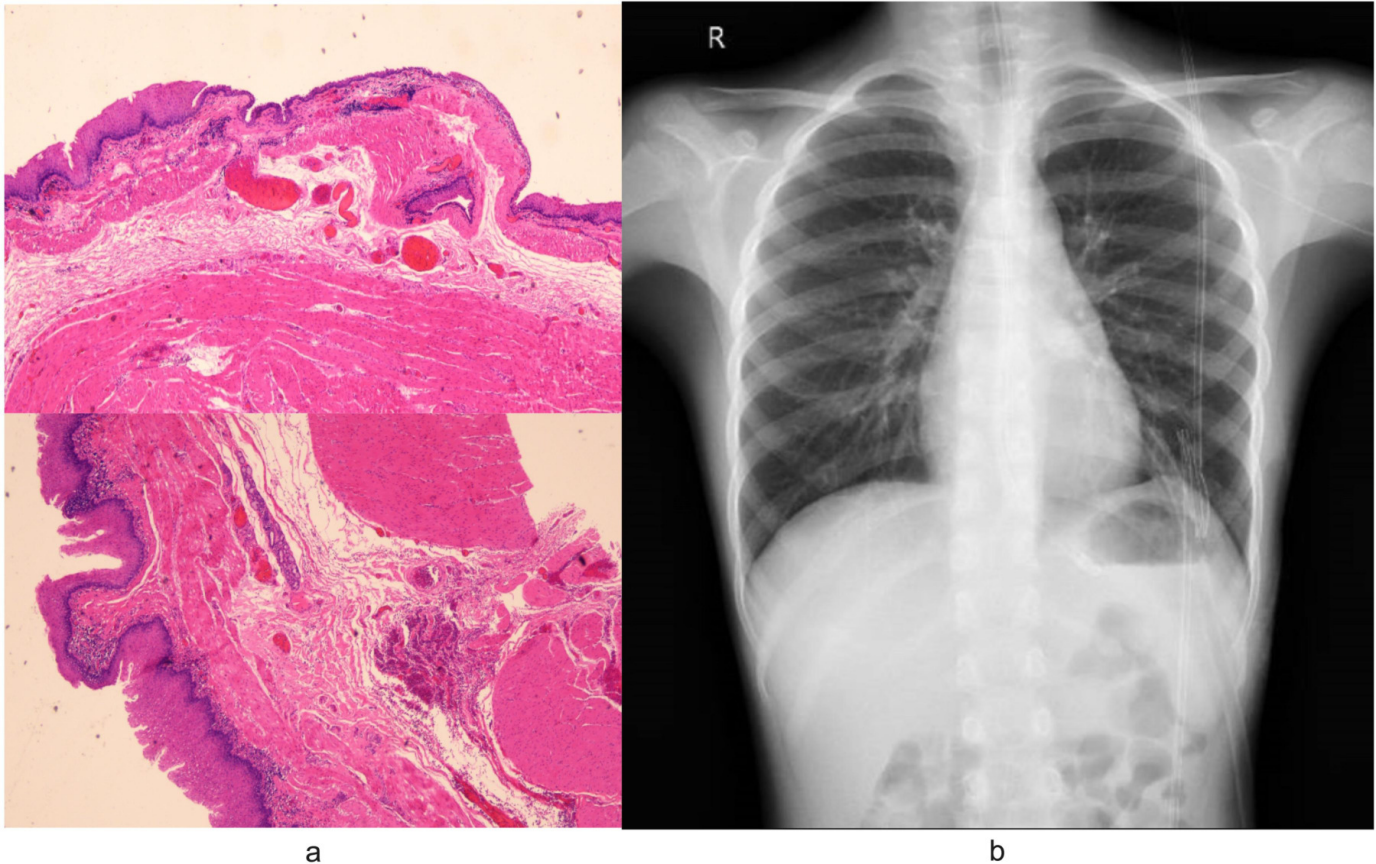
**(a)** Pathological analysis of specimens removed during surgery confirmed the diagnosis of EDC. Microscopic examination revealed characteristic histologic features of the EDC: the cyst wall demonstrated stratified squamous epithelium overlying hyperplastic smooth muscle bundles, focal salivary gland tissue with duct-like structures, and lymphocytic infiltration. **(b)** Postoperative radiography confirmed complete cyst resection.

The surgery was successfully completed without requiring additional ports or conversion to open surgery. The total operative time was 135 min, while the docking time and console time were 15 min and 105 min, respectively. No postoperative complications occurred. Pathological analysis confirmed the diagnosis of EDC (Figure 4a). Postoperative chest radiography demonstrated complete cyst resection (Figure 4b). The patient achieved satisfactory cosmetic outcomes from the minimally invasive approach and was discharged on postoperative day 6. In our case, postoperative pain was systematically assessed using a numerical rating scale (NRS) at 24, 48, and 72 h after surgery. The patient reported NRS scores of 2, 1, and 0, respectively, and required only acetaminophen for analgesia with no need for opioid medications. While we did not have direct comparative data with conventional thoracoscopic surgery in this single case, this pain profile aligns with previously reported outcomes of robotic-assisted thoracic procedures in pediatric patients, which have demonstrated comparable or lower pain scores compared to conventional thoracoscopy (9, 10). This may be attributed to the robotic platform's enhanced precision in dissection and tissue handling, which minimizes collateral trauma despite the similar trocar size.

## 4 Discussion

An esophageal cyst qualifies as a duplication cyst only if it satisfies all three criteria: (1) location within or adherence to the esophageal wall; (2) double muscular layer encapsulation; and (3) epithelial lining comprising squamous, columnar, cuboidal, pseudostratified, or ciliated cells (11). Clinical manifestations are diverse; the predominant presenting symptoms include dysphagia, epigastric discomfort, and retrosternal pain (3). In the present case, the patient presented with a one-week history of intermittent left-sided chest pain, bloating, and nausea. CT, chest radiography, and US are critical for preoperative diagnosis of EDC (12). In our case, the PET/CT scan was informative, as it identified a paraspinal cystic lesion in the left lower lobe with absent metabolic activity. Intraoperatively, the lesion was confirmed to be cystic, consistent with the expected features of a duplication cyst. Surgical excision serves as the definitive treatment for esophageal cysts while simultaneously enabling histopathological confirmation through examination of the resected specimen (11, 12). Minimally invasive surgical techniques have been extensively utilized in pediatric surgery, with research efforts in this area growing over the past three decades—including a pivotal “golden decade” in the early 21st century (13, 14). In addition, RATS offers significant advantages in pediatric practice: accelerated postoperative recovery, a shortened surgeon learning curve, and superior cosmesis, which collectively drives its clinical adoption (5). More deeply, for a pediatric EDC with dense adhesions to critical structures, RATS provided superior technical advantages over conventional thoracoscopy. The robotic system's 3D high-definition visualization enabled precise identification of anatomical planes, while its articulated instruments with tremor filtration allowed for meticulous dissection and stable, tension-free suturing of the esophageal defect. These capabilities minimized the risk of injury and postoperative complications, contributing to a favorable outcome with minimal pain and early discharge, underscoring RATS as a safe and effective approach for complex pediatric mediastinal anomalies (5, 15).

A review of published literature on RATS in pediatric patients indicates this approach has proven to be a safe and effective method for managing EDC (5, 15, 16). However, reports on the application of RATS for pediatric EDC remain scarce in China. In this context, we present a successful case managed via RATS, thereby reinforcing the efficacy and feasibility of this approach. Intraoperatively, the mediastinal cyst contained chylous fluid with dense adhesions to the esophagus, thoracic aorta, and left lower lobe. Given the significant risk of mucosal violation during dissection, meticulous care was taken to prevent accidental injury. The cyst was carefully separated from the esophageal wall by dividing superficial muscle fibers while preserving mucosal integrity. We employed interrupted suturing to enhance blood circulation and healing through inter-suture spacing. This technique concurrently reduces infection risk by improving wound ventilation, demonstrating clinical efficacy.

The application of RATS in children presents two notable limitations: First, the Da Vinci system incurs higher hospitalization costs and requires longer setup times compared to open approaches, though these disparities may diminish with surgical proficiency and institutional experience. Second, pediatric surgery lacks standardized robotic training protocols, creating variability in skill acquisition.

## 5 Conclusion

In conclusion, we present a successful case of an 8-year-old child with EDC managed via RATS. As far as we know, this represents an exceptionally rare report of EDC treatment using the Da Vinci robotic system in a pediatric patient within China. Given risks of complications and malignant transformation, surgical intervention remains indicated regardless of symptomatology. This case demonstrates the safety and feasibility of RATS for treating pediatric EDC, though further development of pediatric specific instruments and long-term outcome studies are warranted for broader validation.

## 6 Patient perspective

The patient's family expressed relief and satisfaction with the appropriate surgical intervention and subsequent recovery.

## Data Availability

The original contributions presented in the study are included in the article/Supplementary Material, further inquiries can be directed to the corresponding author.

## References

[1] Sun C-F Chen C-H Ke P-Z Ho T-L Lin C-H. Esophageal duplication cyst presenting with stridor in a child with congenital pulmonary airway malformation. Medicine (Baltimore). (2019) 98:e16364. 10.1097/MD.0000000000016364 31305433 PMC6641662

[2] Whitaker JA Deffenbaugh LD Cooke AR. Esophageal duplication cyst. Case report. Am J Gastroenterol. (1980) 73:329–32. 7416128

[3] Wahi JE Safdie FM. Esophageal duplication cysts: a clinical practice review. Mediastinum. (2023) 7:1–1. 10.21037/med-22-33 36926292 PMC10011867

[4] Parikh D Short M. “Esophageal Duplication Cyst.,” Pediatric Surgery. Berlin, Heidelberg: Springer Berlin Heidelberg (2017). p. 1–14. 10.1007/978-3-642-38482-0_50-1

[5] Wahi JE Safdie FM. Robotic-assisted approach to esophageal duplication cysts. Annals of Esophagus. (2024) 7:13–13. 10.21037/aoe-24-14

[6] Jacobson JC Pandya SR. Pediatric robotic surgery: an overview. Semin Pediatr Surg. (2023) 32:151255. 10.1016/j.sempedsurg.2023.151255 36736161

[7] Damani T Ballantyne G. Robotic foregut surgery. Surg Clin North America. (2020) 100:249–64. 10.1016/j.suc.2019.11.002 32169179

[8] Kerwan A Al-Jabir A Mathew G Sohrabi C Rashid R Franchi T Revised surgical CAse REport (SCARE) guideline: an update for the age of artificial intelligence. Premier J Sci. (2025) 10:100079. 10.70389/PJS.100079

[9] Aithal S Sinha A Pathak M. Robotic assisted thoracoscopic surgery in children: a narrated review. J Pediatr Endosc Surg. (2024) 6:107–14. 10.1007/s42804-023-00210-y

[10] Wei S Huang T Liang L Gao Y Zhang J Xia J Efficacy of Da Vinci robot-assisted thoracoscopic surgery in children with congenital cystic adenomatiod malformation. J Pediatr Surg. (2024) 59:1458–62. 10.1016/j.jpedsurg.2024.02.034 38553403

[11] Sodhi KS Saxena AK Narasimha Rao KL Singh M Suri S. Esophageal duplication cyst. Pediatr Emerg Care. (2005) 21:854–6. 10.1097/01.pec.0000190236.50728.0d 16340764

[12] Kunisaki C. Esophageal duplication cyst. Ann Laparosc Endosc Surg. (2022) 7:31–31. 10.21037/ales-22-43

[13] Shu B Zhang S Gao J Wang L Wang X. Robotic-assisted pyeloplasty in a five-day-old infant with severely infected hydronephrosis: the very young case report and review of literature. Int J Surg Case Rep. (2025) 132:111478. 10.1016/j.ijscr.2025.111478 40499443 PMC12182382

[14] Shu B Feng X Martynov I Lacher M Mayer S. Pediatric minimally invasive surgery—a bibliometric study on 30 years of research activity. Children. (2022) 9:1264. 10.3390/children9081264 36010154 PMC9406539

[15] Obasi PC Hebra A Varela JC. Excision of esophageal duplication cysts with robotic-assisted thoracoscopic surgery. JSLS. (2011) 15:244–7. 10.4293/108680811X13071180406961 21902985 PMC3148881

[16] Ringley C Bochkarev V Oleynikov D. Esophageal duplication cyst–a guest case in robotic and computer-assisted surgery from the University of Nebraska Medical Center. MedGenMed. (2006) 8:25. 17415307 PMC1868358

